# A single-centre study on abnormal antinuclear antibodies in children caused by intravenous infusion of gamma globulin

**DOI:** 10.3389/fimmu.2024.1410661

**Published:** 2024-07-18

**Authors:** Li Xu, Juan Zhou, Yu Zhang, Yating Wang, Xin Yan, Li Wang, Xuemei Tang, Chong Luo

**Affiliations:** ^1^ Department of Rheumatology and Immunology, Children's Hospital of Chongqing Medical University, National Clinical Research Center for Child Health and Disorders, Ministry of Education Key Laboratory of Child Development and Disorders, Chongqing, China; ^2^ Chongqing Key Laboratory of Child Rare Diseases in Infection and Immunity, Chongqing, China

**Keywords:** children, antinuclear antibody, anti-SSA, anti-Ro52, anti-Mi2, intravenous infusion of gamma globulin

## Abstract

**Objective:**

To clarify the impact of intravenous infusion of gamma globulin (IVIg) on antinuclear antibodies (ANAs) in children.

**Methods:**

A retrospective analysis was performed on the data of children with nonspecific autoantibody-related diseases whose antinuclear antibody (ANA) and autoantibody profiles were detected in our hospital from January to March 2022. A total of 108 patients with a clear history of IVIg infusion within 28 days composed the IVIg group, and 1201 patients without a history of IVIg infusion composed the non-IVIg group.

**Results:**

All patients in the IVIg group had either positive ANAs or positive autoantibodies. Anti-SSA, anti-Ro52 and anti-AMA Mi2 were the top three autoantibodies in the IVIg group. The proportions of patients who were positive for either of these three autoantibodies in the IVIg group were significantly greater than those in the non-IVIg group (all P<0.5). Spearman correlation analysis revealed that the signal intensities of anti-SSA and anti-Ro52 were negatively correlated with the number of days of ANA detection after IVIg infusion (P<0.05). Multiple logistic analyses revealed that a greater total dosage of IVIg, greater IVIg per kilogram of body weight, and fewer ANA detection days after IVIg infusion were independent risk factors for positive anti-SSA and anti-Ro52 results.

**Conclusions:**

It is recommended that if rheumatic diseases are suspected, ANA detection should be carried out beforeIVIg infusion. But for patients who are positive for at least one of these three autoantibodies after IVIg infusion, doctors should first consider adoptive antibodies.

## 1 Introduction

With the deepening understanding of rheumatic diseases, an increasing number of paediatric patients with rheumatic diseases have been diagnosed in recent years (1). Due to the diverse clinical manifestations of rheumatic diseases, paediatricians often complete ANA detection for patients with unexplained fever or multisystem involvement. Autoantibodies are very important serological markers of rheumatic diseases (2, 3). Some specific autoantibodies, such as anti-dsDNA for systemic lupus erythematosus (SLE), anti-SSA for Sjögren’s syndrome (SS), and anti-U1nRNP for mixed connective tissue disease, are important in the classification criteria for autoimmune diseases (4, 5). However, positive ANA or specific autoantibodies do not necessarily indicate rheumatic diseases. For example, ANA and some autoantibodies can also be positive in viral infections (hepatitis C virus, parvovirus), tuberculosis infections, parasitic infections (schistosomiasis), and tumours (6–8). In addition, approximately 5% of healthy people may also have a low titre of ANA (9).

Intravenous infusion of immunoglobulin (IVIg) is a liquid or freeze-dried powder preparation in which the main component is polyclonal immunoglobulin G (IgG), which is isolated from the plasma of more than 1,000 healthy blood donors. IVIg was originally used as an alternative treatment for patients with primary immunoglobulin deficiency. In recent years, IVIg has become increasingly important for the treatment of autoimmune/inflammatory diseases and severe infectious diseases in children (10, 11). Because of its effectiveness and safety, paediatric patients who are difficult to diagnose or are in critical condition may have already received IVIg infusion before a clear diagnosis is made. Sometimes, ANA may be detected after the infusion of IVIg. Therefore, some patients who are positive for ANA or autoantibodies but without other obvious evidence of rheumatic disease usually have a history of IVIg infusion before ANA detection. Some of these positive autoantibodies are specific for certain rheumatic diseases, which may lead to difficulties in making diagnoses by paediatricians.

We considered that IVIg infusion could transfer IgG autoantibodies to the recipients, which led to positive ANA detection in the recipients. Previous studies have shown that anti-SSA is present in IVIg products and in blood donors without clinical symptoms, which makes IVIg replacement interfere with ANA and ENA serology through the passive transfer of autoantibodies (12–14). Renate’s study also showed that discoid erythema occurred in a common variable immunodeficiency (CVID) patient receiving regular IVIg replacement therapy, which may be related to the transfer of anti-SSA by infused IVIg (12). At present, there are no data about the ANA and autoantibody profiles of children after IVIg infusion worldwide. This study aimed to analyse the ANA of patients who were not diagnosed with autoantibody-specific rheumatic diseases after IVIg infusion in our hospital to assist clinicians in evaluating positive ANA or autoantibodies for diagnosis after IVIg infusion.

## 2 Materials and methods

### 2.1 Material

There were 3683 patients with nonspecific autoantibody-related diseases who underwent ANA and autoantibody profile detection at the Children’s Hospital of Chongqing Medical University between January and March 2022. There were 108 patients with a clear history of IVIg infused within 28 days before ANA detection in the IVIg group. A total of 1201 patients without a history of IVIg infused before ANA detection were randomly selected as the non-IVIg group. The basic information and diagnosis of both groups were recorded, and the ANA detection time after IVIg infusion and the dosage of IVIg in the IVIg group were recorded. The proportions of positive ANA or ANA‐specific antibodies in both groups were compared. Moreover, ITP patients from the IVIg group and non-IVIg group were also screened for comparisons of ANA and certain specific antibodies. The relationships of ANA detection days after IVIg infusion, the dosage of IVIg and ANA positivity or ANA‐specific antibodies in the IVIg group were analysed.

All patients were excluded if they had specific autoantibody-related rheumatic diseases, such as systemic lupus erythematosus, Sjögren’s syndrome, undifferentiated connective tissue disease, mixed connective tissue disease, systemic scleroderma, juvenile dermatomyositis, or protozoan idiopathic biliary cirrhosis and so on. Patients with rheumatic diseases without a clear relationship with specific autoantibodies, such as Henoch-Schönlein purpura, Kawasaki disease, autoimmune haemolytic anaemia, immune thrombocytopenic purpura, and juvenile idiopathic arthritis, were included. Infectious and noninfectious diseases, such as sepsis, pneumonia, neurological diseases, renal diseases and cardiovascular system diseases, were also included. This study was approved by the Ethics Committee of the Children’s Hospital of Chongqing Medical University.

Because some patients were infused with IVIg in other hospitals, we did not know the manufacturers of the IVIg. However, all IVIg was liquid, and the IVIg used in our hospital came from three different manufacturers. Therefore, there were many manufacturers of IVIg in this research.

### 2.2 Methods

Serum ANA titres were detected by indirect immunofluorescence (IIF), and 15 other ANA‐specific IgG antibodies were detected by Euroline immunoassays. ANAs were detected by IIF in HEp‐2 cells according to the manufacturer’s instructions (Euroimmun AG). ANA titres >1:80 were considered positive. Euroline ANAs (IgG) were used to qualitatively detect 15 IgG antibodies (anti‐nRNP, anti‐Sm, anti‐SSA, anti‐Ro52, anti‐SSB, anti‐Scl‐70, anti‐PM-Scl, anti‐Jo1, anti‐CENP B, anti‐dsDNA, anti‐PCNA, anti‐nucleosome, anti‐Histone, anti‐ribosomal P protein, and anti‐AMA M2 (anti-Mi2)) in human serum. Anti-SSA was equivalent to anti-Ro60, not included anti-Ro52. The serum for the ANA profile (IgG) was diluted 1:101.

All the reagents used were produced by the German Oumeng Medical Detection and Diagnostics Co., Ltd. According to the degree of colouration, the detection signal intensity was divided into negative (-), suspicious positive (±), generally positive (+), positive (++), and strongly positive (+++). The final colour film strip results were automatically interpreted using the EUROBlot Master and EUROBline S-can systems. We considered negative (-) and suspicious positive (±) negative results in this study.

### 2.3 Statistical methods

All the statistical analyses were performed using SPSS 25.0. Fisher’s test was used for categorical variables. The number of days of ANA detection after IVIg infusion and the dosage of IVIg were used as continuous variables. T-test was used to compare mean values between normally distributed data. Spearman correlation analysis was performed to analyse correlations between positive ANA or ANA-specific antibodies and other variables. Multivariate logistic regression analysis was further performed to analyse the correlations between the number of detection days after IVIg infusion, the dosage of IVIg and certain ANA-specific antibodies. The signal intensity of anti-SSA was analysed as a categorical variable by (-) and (+), and the signal intensity of anti-Ro52 and anti-Mi2 was analysed by (-), (+), and (++). P<0.05 was considered to indicate a statistically significant difference.

## 3 Results

### 3.1 Baseline information

In the IVIg group (108 patients), 32 patients were ultimately diagnosed with infectious diseases, and 76 patients were diagnosed with noninfectious diseases, including ITP (27 patients), Kawasaki disease, disseminated encephalomyelitis, central nervous system demyelination, and nephrotic syndrome. In the non-IVIg group (1201 patients), 233 patients had infectious diseases, and 968 patients had noninfectious diseases, including ITP (116 patients) and other diseases, as IVIg group (**Table 1**). The median age of the patients in the IVIg group was 3.08 years [1 day–14.92 years]. The median duration from IVIg infusion to ANA detection in 108 patients was 2 days [0.5~26 days], the median total dosage of IVIg was 20g [2.5–115g], and the median dosage of IVIg was 1.55 g/kg body weight [0.14~4 g/kg].

**Table 1:** Comparison of basic information between the IVIg group and the non-IVIg group.

	IVIg group	non-IVIg group	t/Fisher’s	*P* value
average age (year)	4.35 ± 3.87	6.39 ± 4.23	4.85	**<0.05**
male: female	61:47	665:536	–	0.6290
infection: noninfection	32:76	233: 968	–	0.1634
positive ANA or specific antibodies n (%)	108(100%)	191(15.89%)	–	**<0.0001**
positive ANA n (%)	55(50.93%)	120(9.99%)	–	**<0.0001**
positive ANA-specific antibodies n (%)	103(95.37%)	87(7.24%)	–	**<0.0001**

Data are means ± standard deviation. Bold statistical significance.

### 3.2 ANAs and ANA-specific antibodies in the IVIg group and non-IVIg group

#### 3.2.1 Antinuclear antibodies

All of the patients in the IVIg group were positive for ANA or ANA-specific antibodies. There were 55 patients (50.93%) with positive ANAs. In the non-IVIg group, 191 patients (15.9%) had positive ANAs or specific autoantibodies, and 120 patients (9.99%) had positive ANAs (**Table 1**). In the IVIg group, the proportion of females with ANA positivity was similar to that in the male subgroup (**Table 2**). In the non-IVIg group, the proportion of females with ANA positivity was greater than that of males with ANA positivity (P<0.05).

**Table 2:** Distribution of the ANA‐positive population by sex.

positive ANA	female n(%)	male n(%)	Fisher’s	*P* value
IVIg group	21/47(44.68%)	34/61(55.74%)	–	0.3319
non-IVIg group	68/536(12.69%)	52/665(7.82%)	–	**0.0065**

Bold statistical significance.

#### 3.2.2 ANA-specific antibodies

There were no patients who were positive for anti-Sm, anti-CENP B, anti-dsDNA, anti-nucleosome, and anti-ribosomal P protein in either group. In the IVIg group, the three most positive antibodies were anti-Ro52, anti-Mi2 and anti-SSA (**Table 3**). The proportion of patients who were positive for anti-Ro52 was the highest, with 95 patients (87.96%), followed by 57 patients who were positive for anti-Mi2 (52.78%) and 42 patients who were positive for anti-SSA (38.89%). The proportions of patients who were positive for these three antibodies were significantly greater in the IVIg group than in the non-IVIg group separately (all P <0.05). There were 30 patients (27.78%) who were simultaneously positive for anti-SSA, anti-Ro52 and anti-Mi2 in the IVIg group, but there were no patients in the non-IVIg group (P<0.05). The three most common positive antibodies in the non-IVIg group were anti-PM-Scl (17 patients; 1.42%), anti-nRNP/Sm (11 patients; 0.92%), and anti-PCNA (9 patients; 0.75%). There were no significant differences in the proportions of patients who were positive for these antibodies between the IVIg group and the non-IVIg group (all P <0.05).

**Table 3:** Comparison of ANA and antibody levels between the IVIg group and the non-IVIg group.

ANA/ANA-specific antibodies	IVIg group n (%)	non-IVIg group n (%)	Fisher’s	*P* value
ANA	55(50.93%)	120(9.99%)	–	**<0.0001**
speckled pattern	50(46.3%)	78(6.49%)	–	**<0.0001**
cytoplasm particle pattern	33(30.56%)	29(2.41%)	–	**<0.0001**
nucleolar pattern	2(1.85%)	15(1.24%)	–	0.6449
the other patterns	2(1.85%)	14(1.17%)	–	0.3856
anti-nRNP/SM	3(2.78%)	11(0.92%)	–	0.1019
anti-SSA	42(38.89%)	3(0.25%)	–	**<0.0001**
anti-Ro52	95(87.96%)	6(0.50%)	–	**<0.0001**
anti-SSB	1(0.93%)	1(0.08%)	–	0.1583
anti-Scl70	1(0.93%)	6(0.50%)	–	0.4535
anti-PM-SCL	2(1.85%)	17(1.42%)	–	0.6662
anti-Jo1	0(0%)	7(0.58%)	–	–
anti-PCNA	1(0.93%)	9(0.75%)	–	0.5786
anti-histone	2(1.85%)	12(1.00%)	–	0.3235
anti AMA-Mi2	57(52.78%)	4(0.33%)	–	**<0.0001**

Bold statistical significance.

#### 3.2.3 ANAs and ANA-specific antibodies from patients with ITP

To completely exclude the effects of different diseases on ANA detection, patients with the same disease from the two groups were selected for analysis of differences in anti-Ro52, anti-Mi2 and anti-SSA. There were 27 patients with a definite diagnosis of immune thrombocytopenia (ITP) in the IVIg group and 116 patients with ITP in the non-IVIg group. There were no differences of age, sex and nadir platelet counts between these two groups (all P>0.05). There were 53 patients in non-IVIg group with IVIg treatment only, and 32 patients with glucocorticoids treatment only, 21 patients with both IVIg and glucocorticoids treatment, 10 patients with neither IVIg nor glucocorticoids treatment. And 9 patients in IVIg group received both IVIg and glucocorticoids treatment. There were no differences of proportions of the patients who received both IVIg and glucocorticoids treatment between these two groups(P>0.05). And there were no patients using other immune modulating treatment in these two groups. In the IVIg group, the three most positive antibodies were still anti‐Ro52, anti-AMA M2, and anti‐SSA. The proportions of patients who were positive for anti-Ro52, anti-AMA Mi2, and anti-SSA were significantly greater in the IVIg group than in the non-IVIg group separately (all P <0.05) (**Table 4**).

**Table 4:** Comparison of ANA and antibodies between ITP patients in the IVIg group and those in the non-IVIg group.

	IVIg group n (%)	non-IVIg group n (%)	t/Fisher’s	*P* value
age(year)	3.26 ± 4.13	4.01 ± 3.91	-0.885	0.377
Female	10(37.04%)	45(38.79%)	–	>0.9999
PLT(×10^9^/L)	9.85 ± 7.50	10.34 ± 7.13	-0.315	0.753
IVIg+glucocorticoids	9	21	–	0.113
ANA	13(48.15%)	13(12.21%)	–	**<0.0001**
speckled pattern	10(37.04%)	10(8.62%)	–	**0.0006**
cytoplasm particle pattern	7(25.93%)	3(2.59%)	–	**0.0003**
anti-SSA	11(40.74%)	1(0.86%)	–	**<0.0001**
anti-Ro52	22(81.48%)	2(1.72%)	–	**<0.0001**
anti AMA-Mi2	14 (51.85%)	0(0.0%)	–	**<0.0001**

Data are means ± standard deviation. Bold statistical significance.

### 3.3 Correlations between ANA/ANA-specific antibodies and other variables in IVIg group

There was no correlation between IVIg per kilogram of body weight and age. The total dosage of IVIg was positively correlated with age (r=0.621, P<0.05) and weight (r=0.620, P<0.05). In the IVIg group, the signal density of all positive anti-SSA signals was (+); the signal density of positive anti-Ro52 signals was 38 (++) and 57 (+); and the signal density of positive anti-Mi2 signals was 35 (++) and 22 (+). Spearman correlation analysis revealed that the levels of anti-SSA and anti-Ro52 were negatively correlated with the number of detection days after IVIg infusion (P<0.05). Anti-Mi2 was negatively correlated with the number of days after IVIg infusion, but the difference was not significant (P>0.05). The anti-Ro52 concentration was positively correlated with the total dose of IVIg (P<0.05). Moreover, anti-SSA was correlated with ANA, anti-Ro52 and anti-Mi2 (all P values <0.05) (**Table 5**).

**Table 5:** Spearman correlation analysis of ANA/ANA-specific antibodies and other variables.

		Sex	age	Wt	detection days	total IVIg	IVIg/kg(Wt)	ANA	anti-Ro52	anti-Mi2
ANA	r	-0.110	0.139	0.155	-0.95	0.183	0.107	–	0.079	0.001
	*P*	0.259	0.15	0.110	0.326	0.058	0.272	–	0.419	0.992
anti-SSA	r	-0.87	0.145	0.137	-0.455	0.165	0.085	0.327	0.563	0.353
	*P*	0.369	0.134	0.158	**0.000**	0.087	0.382	**0.001**	**0.000**	**0.000**
anti-Ro52	r	-0.167	0.222	0.231	-0.379	0.268	0.144	–	–	0.126
	*P*	0.085	**0.021**	**0.016**	**0.000**	**0.005**	0.137	–	–	0.195
anti-Mi2	r	0.041	0.026	0.010	-0.158	0.085	0.063	–	–	–
	*P*	0.672	0.786	0.918	0.103	0.381	0.514	–	–	–

Bold statistical significance.

### 3.4 Multivariate logistic regression analysis of IVIg dosage and ANA detection days after IVIg infusion with positive antibodies

Anti-SSA, anti-Ro52 and anti-Mi 2 were the dependent variables (0 as negative, 1=+, 2=++), and IVIg dosage (total dosage or IVIg per kilogram of body weight) and detection days after IVIg infusion were the independent variables. A higher total dosage of IVIg, higher IVIg per kilogram of body weight, and a shorter detection day after IVIg infusion were found to be independent risk factors for positive anti-SSA or positive anti-Ro52 (**Tables 6**, **7**). The total dosage of IVIg or IVIg per kilogram of body weight and detection days after IVIg infusion were not independent risk factors for positive anti-Mi2 (**Tables 6**, **7**).

**Table 6:** Multivariate logistic regression analysis of total dosage, days of ANA detection and levels of antibodies.

		OR	95% CI		*P* value
anti-SSA	total dosage of IVIg	1.026	1.004	1.049	**0.023**
	detection days after IVIg infused	-0.752	-0.644	-0.877	**<0.0001**
anti-Ro52	total dosage of IVIg	1.042	1.019	1.066	**<0.0001**
	detection days after IVIg infused	-0.872	-0.815	-0.934	**<0.0001**
anti-Mi2	total dosage of IVIg	1.005	0.990	1.021	0.491
	detection days after IVIg infused	-0.965	-0.908	-1.023	0.233

OR, odds ratio; 95% CI, 95% confidence interval. Bold statistical significance.

**Table 7:** Multivariate logistic regression analysis of IVIG/kg(Wt), days of ANA detection and levles of antibodies.

		OR	95% CI		*P* value
anti-SSA	IVIg/kg(Wt)	1.834	1.001	3.360	**0.049**
	detection days after IVIg infused	-0.741	-0.630	-0.870	**<0.0001**
anti-Ro52	IVIg/kg(Wt)	1.831	1.080	3.105	**0.025**
	detection days after IVIg infused	-0.876	-0.819	-0.937	**<0.0001**
anti-Mi2	IVIg/kg(Wt)	1.324	0.818	2.143	0.253
	detection days after IVIg infused	-0.960	-0.903	-1.020	0.187

OR, odds ratio; HR, hazards ratio; 95% CI, 95% confidence interval.

Bold statistical significance.

### 3.5 Longitudinal ANAs and ANA-specific antibodies of a small group of patients from IVIg group

A total of 20 patients from the IVIg group had multiple ANA tests in our hospital. Among them, 7 patients had tested for ANAs before this study, and all of them had negative anti-SSA, anti-Ro52 and anti-Mi2, but after IVIg infusion in this study, 6 of them had at least one of the three autoantibodies positive. There were 11 patients with repeated ANA tests after this study, 10 of them had at least one of the three autoantibodies positive in this study and only one of them had anti-Ro52 positive when they repeated ANA tests later. There were 2 patients with ITP had ANA tests both before and several months after this study, and the three autoantibodies were all negative, but in this study they had at least two of the three autoantibodies positive.

## 4 Discussion

IVIg is a polyclonal immunoglobulin extracted from the plasma of thousands of healthy people. It contains approximately 50 g/L protein, of which the gamma globulin content is no less than 95% (15). A previous study revealed that a CVID patient who regularly received IVIg were positive for ANA and anti-SSA. It was also found that the proportion of anti-SSA antibodies in the blood of healthy donors without any overt autoimmune features was approximately 0.69%, and 0.05% of donors had very high (greater than 10,000 U/ml) anti-SSA titres (12). To further study the effect of IVIg infusion on ANA detection in paediatric patients, we analysed ANA and ANA-specific antibodies in the IVIg group and non-IVIg group.

In this study, all of the patients in the IVIg group had positive ANA or ANA-specific antibodies. The speckled pattern and cytoplasm particle pattern were the most common patterns of ANA in the IVIg group and non-IVIg group. The percentage of ANA-positive individuals in the non-IVIg group reached 9.99%, 12.51% of which were females (66/536), which was significantly greater than the 7.37% of which were males (49/665). These data were similar to the percentages of ANA-positive individuals in healthy people reported in the Chinese literature (14.1%), of which 19.05% were females (2376/12470), which is greater than the 9.04% among males (1143/12640) (16). However, the proportion of ANA-positive individuals was significantly greater in the IVIg group than in the non-IVIg group, and there was no difference in the proportion of ANA-positive individuals between males and females, which was different from that in the non-IVIg group. These results indicated that IVIg infusion led to a greater proportion of ANA-positive patients in the IVIg group by transferring autoantibodies to the recipients.

To further study the effect of IVIg infusion on ANA-specific antibodies, 15 autoantibodies were analysed. The top three autoantibodies detected in the IVIg group were anti-Ro52 (87.96%), anti-Mi2 (52.78%), and anti-SSA (38.89%), which were the same as the top three antibodies detected in 1489 ANA-positive people in a study of 25110 healthy adults with anti-Ro52 (212 cases, 14.24%), anti-M2 (189 cases, 12.69%) and anti-SSA (144 cases, 9.67%) (16). However, the proportions of patients who were positive for either of these autoantibodies were much greater than those in the healthy adult and non-IVIg groups. The average age of non-IVIg group was older than that of IVIg group, therefore, ITP patients who had no differences of age and disease between those two groups were compared, and the same results were gotten. Anti-SSA, anti-Ro52 and anti-Mi2 were not among the three most common antibodies in the non-IVIg group, suggesting that the distribution of autoantibodies in the plasma of adults may differ from that in the plasma of children. Shome’s study showed that the number of unique IgG autoantibodies in healthy individuals increased with age from infancy to adolescence and then plateaued (17). However, while the response to infectious agents (and possibly vaccines) might contribute to autoantibodies through molecular mimicry, this mechanism does not appear to continue to result in the accumulation of autoantibodies throughout life (17). The plasma donors were all adults, which can explain why the top three antibodies were the same between IVIg group patients and healthy adults. Although a previous study showed that one patient who received IVIg developed discoid lupus erythematosus, the abovementioned patients in the IVIg group had no clinical manifestations of rheumatic disease. It is speculated that the risk of IVIg infusion leading to the development of clinical phenotypes of rheumatic diseases is very low.

Anti-SSA was named after the discovery of the A antigen in Sjogren syndrome patients. It was previously believed that the target antigens of anti-SSA include two proteins with molecular weights of 60 kDa and 52 kDa. Ro60 and Ro52 are the same macromolecular complex, so the anti-SSA system included anti-SSA/Ro60 and anti-SSA/Ro52. Chan’s research showed that the Ro60 and Ro52 antigens are proteins encoded by two different cDNAs. The natural SSA antigen only has Ro60 but not Ro52 (18). Anti-Ro60 and anti-Ro52 do not belong to the same antibody system and have different clinical significance (19). In this study, anti-SSA was equivalent to anti-natural SSA or anti-Ro60. Anti-SSA is one of the few autoantibodies that can cause immunopathological injury. It can lead to neonatal lupus or photoallergic dermatitis (20). Anti-SSA is present in many rheumatic diseases, such as 20~60% of SLE patients, 40~95% of SS patients, and 95~100% of neonatal lupus patients. Because of its importance, we often pay more attention to anti-SSA. Many studies have shown that IVIg contains anti-SSA (12). In this study, we showed that anti-SSA can be tested in recipients by the infusion of IVIg. Its presence in the recipients was also related to the dosage of IVIg and was usually accompanied by positivity for anti-Ro52 or anti-Mi2. Within 28 days, it decayed obviously over time.

Anti-Ro52 has been shown to be involved in the mechanisms of many rheumatic diseases, including SLE, systemic sclerosis, inflammatory myositis and juvenile idiopathic arthritis (21, 22). The proportions of patients with different diseases who are positive for anti-Ro52 antibodies vary greatly. Some studies have shown that it is an independent risk factor for adult dermatomyositis interstitial lung disease or is associated with pulmonary fibrosis and the severity of juvenile dermatomyositis (23, 24). However, most reports indicate that Ro52 is not disease specific. In this study, anti-Ro52 was the most commonly detected antibody after IVIg infusion, with the positive proportion reaching 87.96% of cases, which was greater than that of anti-SSA and anti-Mi2. Moreover, 40% of the positive patients had a signal density of (++). Previous studies have shown that anti-Ro52 and anti-SSA are the most common autoantibodies in the healthy population (14, 16). However, Renate G reported that anti-SSA and anti-Ro52 can be present in IVIg preparations and in apparently healthy donors (12). The results of the present study suggested that IVIg carried a large amount of anti-Ro52, even more so than anti-SSA. Like the presence of anti-SSA, the presence of anti-Ro52 in the recipients was also related to the dosage of IVIg and the detection time after IVIg infusion; within 28 days, the dose also clearly decreased over time.

Antimitochondrial antibodies(AMAs) are autoantibodies directed against mitochondrial inner membrane lipoproteins in the cytoplasm. It is divided into 9 subtypes, among which the M2 subtype is a serological marker of primary biliary cholangitis (PBC) and plays an important role in the diagnosis of PBC (25). PBC is an immune-mediated, progressive, nonsuppurative inflammatory disease of the bile ducts of unknown aetiology. The specificity of anti-AMA Mi2 for PBC is as high as 90% (26). The incidence of PBC in children is very low, and none of the patients in the IVIg group showed signs of cholestasis. The IVIg dosage and number of detection days after IVIg infusion were not independent risk factors for anti-Mi2, which showed that anti-Mi2 may be present longer in the patient’s blood than may anti-SSA and anti-Ro52, and even a lower dosage of IVIg can also lead to positive anti-Mi2 in recipients. Therefore, it took a longer time for the anti-Mi2 antibody to decay.

In this study, ANA patterns did not always coincide with ANA-specific antibodies. The presence of anti-SSA, anti-Ro52 and anti-Mi2 antibodies indicated a speckled pattern. However, in this study, positive anti-SSA or anti-Mi2 results may not always be accompanied by a positive speckled pattern. We believe that the methods of ANA detection and ANA-specific antibody detection differ, and the major challenge and limitation of ANA-IIF testing is that it requires competent and experienced interpreters and is subjective (27), which may explain the above results.

In summary, after IVIg infusion, adoptive autoantibodies can be detected in recipients, which can lead to positive ANA or ANA-specific antibodies. Anti-Ro52, anti-Mi2 and anti-SSA are the top three autoantibodies after IVIg infusion. Within 28 days, the anti-Ro52 and anti-SSA antibodies decreased over time, but the anti-Mi2 antibody was present in the blood of the recipients longer. Combined with the ANAs and ANA-specific antibodies of patients from the IVIg group besides this study, we considered that the autoantibodies conferred by IVIg were detected transiently. It is recommended that if rheumatic diseases are suspected, ANA detection should be carried out before IVIg infusion. However, if patients are at least one of these three antibodies positive after IVIg infusion, doctors should consider adoptive antibodies first, and follow-up visits are also very important.

## Data availability statement

The original contributions presented in the study are included in the article/supplementary material. Further inquiries can be directed to the corresponding author.

## Ethics statement

The studies involving humans were approved by the Ethics Committee of the Children’s Hospital of Chongqing Medical University. The studies were conducted in accordance with the local legislation and institutional requirements. Written informed consent for participation was not required from the participants or the participants’ legal guardians/next of kin in accordance with the national legislation and institutional requirements.

## Author contributions

LX: Conceptualization, Data curation, Formal analysis, Investigation, Methodology, Writing – original draft. JZ: Investigation, Writing – review & editing. YZ: Methodology, Writing – review & editing. YW: Writing – review & editing, Conceptualization. XY: Writing – review & editing, Data curation. LW: Writing – review & editing, Methodology. XT: Supervision, Writing – review & editing, Funding acquisition, Resources. CL: Writing – original draft, Writing – review & editing, Data curation, Investigation, Methodology, Supervision.
